# Emergence of influenza A(H3N2) subclade K in northeast Ohio in autumn 2025

**DOI:** 10.1128/jcm.01813-25

**Published:** 2026-01-27

**Authors:** Xuan Xu, William Hull, David Plunkett, Zheng Jin Tu, Ted M. Ross, Daniel D. Rhoads, Hannah Wang

**Affiliations:** 1 Pathology and Laboratory Medicine Department, Cleveland Clinic 2569, Cleveland, Ohio, USA; 2 Florida Research and Innovation Center, Cleveland Clinic 587918 , Port Saint Lucie, Florida, USA; 3 Microbial Sciences in Health Department, Cleveland Clinic Research, Cleveland Clinic 2569, Cleveland, Ohio, USA; 4 Department of Pathology, Cleveland Clinic Lerner College of Medicine, Case Western Reserve University School of Medicine 12304, Cleveland, Ohio, USA; Vanderbilt University Medical Center, Nashville, Tennessee, USA

**Keywords:** whole-genome sequencing, subclade K, A(H3N2), influenza

## LETTER

A newly emerged influenza A(H3N2) subclade, designated K (formerly J.2.4.1), was identified in June 2025 and rapidly spread worldwide (1–3). This drifted subclade exhibits antigenic divergence from the current northern hemisphere vaccine strain (1, 2). Early observations also indicate that subclade K may be contributing to an unusually early start of the 2025–2026 influenza season in several countries (3). Here, we report the identification of subclade K during clinical validation of a laboratory-developed test for influenza A virus (IAV) subtyping by whole-genome sequencing (WGS) and describe its prevalence in northeast Ohio in autumn 2025.

A convenience cohort of nasopharynx/nasal swab specimens with IAV detected with cycle threshold <31 was sequenced. In brief, the IAV WGS assay uses a previously described multi-segment reverse transcription PCR to generate and amplify viral cDNA for all eight RNA gene segments (4, 5). Fragmented libraries were prepared using Nextera XT DNA library preparation kits (Illumina) and sequenced using MiSeq (Illumina) with paired-end 2 × 150 bp reads. IRMA was used for alignment, assembly, and consensus determination (6), with downstream quality control (QC) metrics integrated using a custom reporting workflow. QC criteria required ≥90% coverage and ≥100× average depth for each gene segment to generate a consensus. Clade/subclade designations were assigned by NextClade using hemagglutinin (HA) consensus sequences (7).

A total of 210 unique specimens collected between December 2024 and November 2025 were tested, and 202 (96.2%) had at least HA passing QC. For the 202 specimens, consensus sequences passing QC had the following count, median coverage, median depth: HA 202, 100%, 6,505×; matrix protein (MP) 194, 100%, 10,910×; polymerase basic 2 (PB2) 190, 100%, 3,365×; neuraminidase (NA) 178, 99.9%, 3,144×; nucleoprotein (NP) 175, 99.9%, 2,701×; polymerase acidic (PA) 172, 100%, 3,012×; nonstructural protein (NS) 164, 100%, 5,693×; and polymerase basic 1 (PB1) 125, 100%, 1,569×.

**Fig 1:**
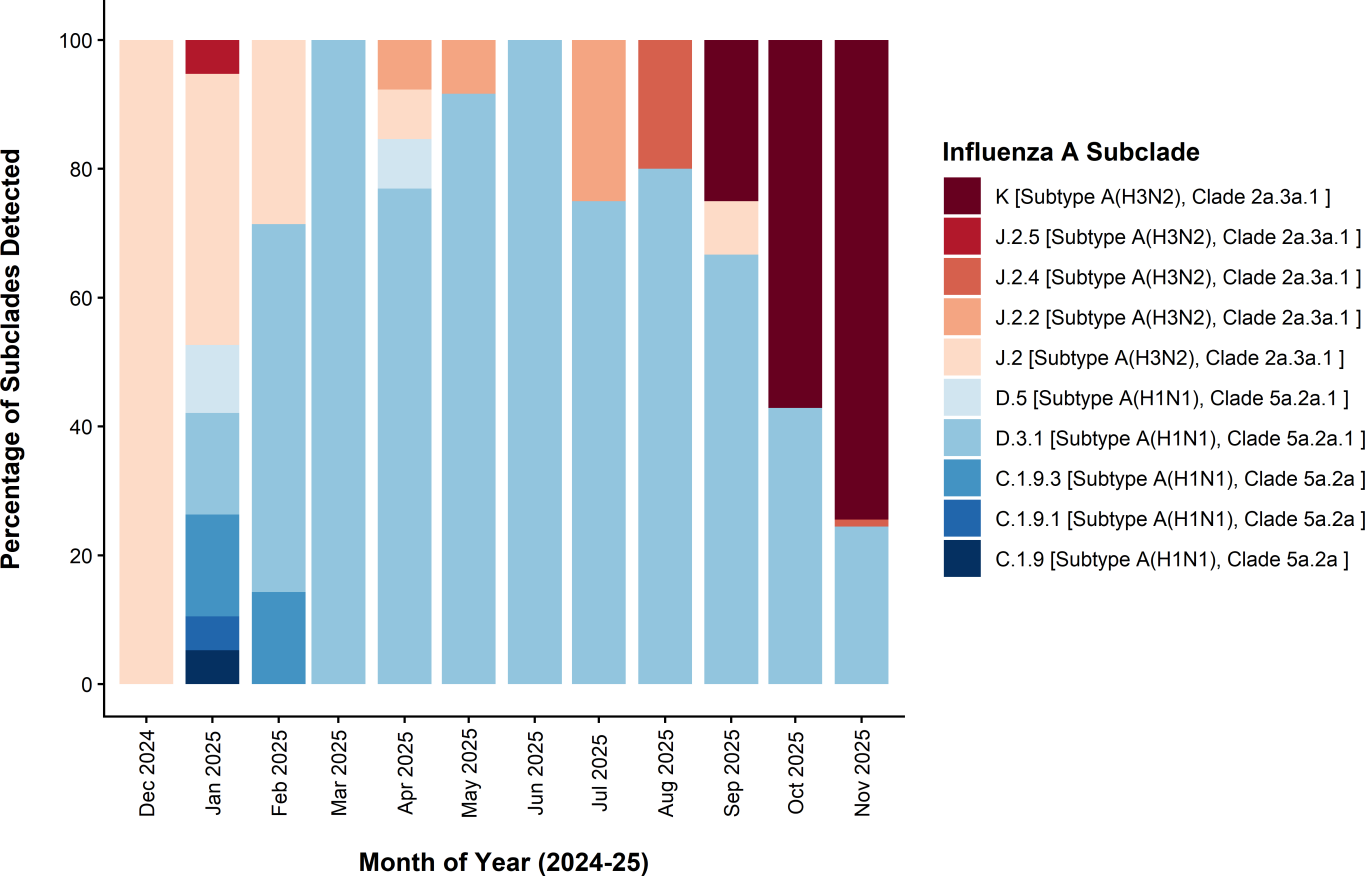
Percentage of sequenced northeast Ohio influenza A virus-positive specimens belonging to each subclade by month of collection. Subclades are designated by color, with those in the red color spectrum belonging to A(H3N2) and those in the blue color spectrum belonging to A(H1N1)pdm09.

**Fig 2:**
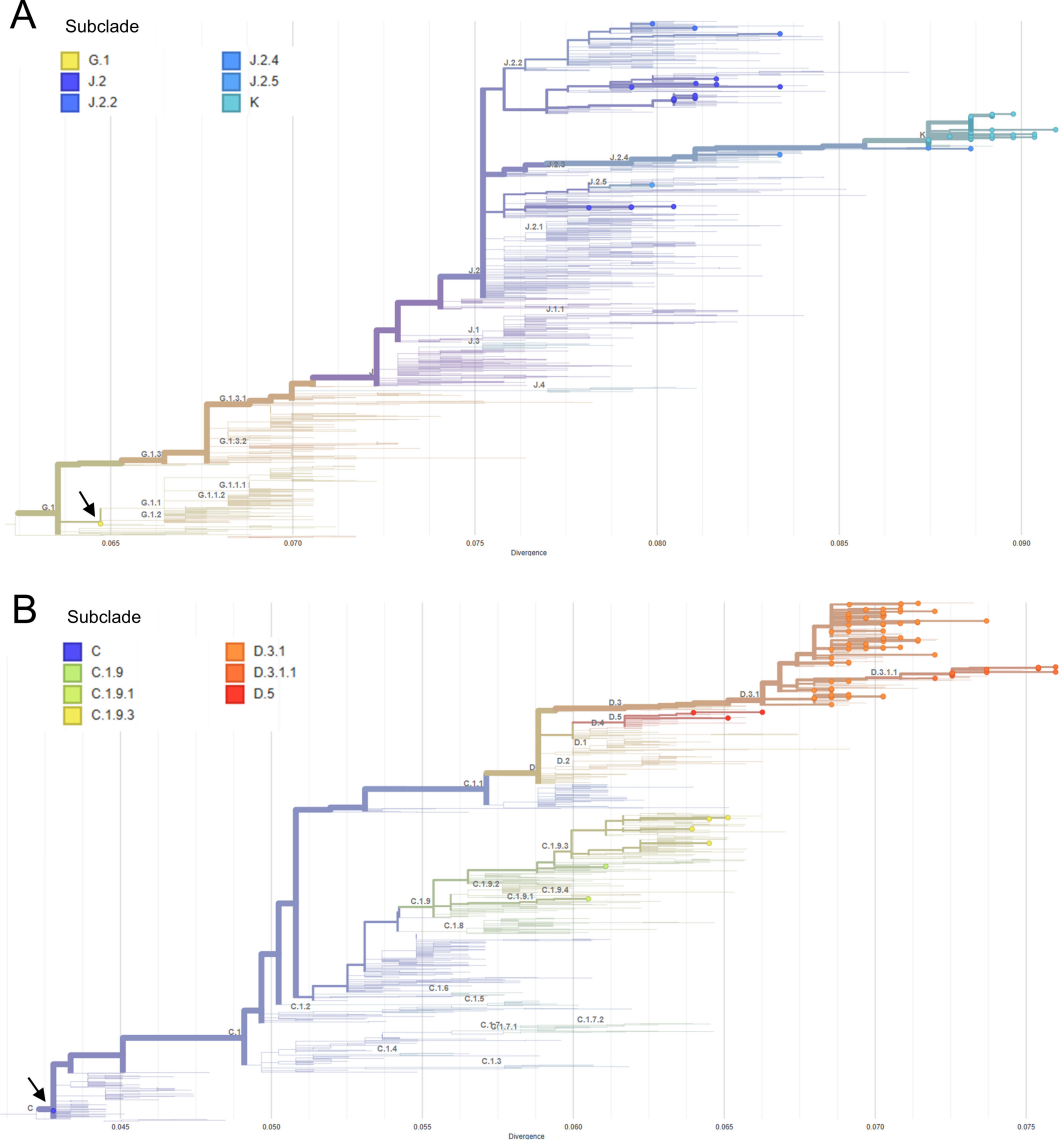
Phylogenetic trees displaying (**A**) 100 influenza A(H3N2) HA segment sequences from this study and (**B**) 102 influenza A(H1N1)pdm09 HA segment sequences from this study in relation to sequences in the Nextstrain global reference tree. Each filled colored circle corresponds to a sequence from this study and is colored by subclade. Black arrows mark the reference sequences used for tree rooting: A/Darwin/6/2021 (EPI1857216) belonging to A(H3N2) clade 2a, subclade G.1 in panel A; A/Wisconsin/588/2019 (MW626062) belonging to A(H1N1) clade 5a.2, subclade C in panel B. Branch length corresponds to nucleotide divergence. Phylogenetic trees were generated in Nextclade v3.18.1 powered using Nextstrain’s Auspice tool (7).

Influenza A(H3N2) subclade K was first identified in early September 2025, coinciding with initial international reports of subclade K expansion. In November 2025, subclade K accounted for 74% (67/90) of sequenced samples (Table 1; Fig. 1). Phylogenetic analysis placed all 74 post-September 2025 A(H3N2) sequences within clade 2a.3a.1 and all 33 post-September 2025 A(H1N1)pdm09 sequences within clade 5a.2a.1 (Fig. 2A and B). Among the A(H3N2) sequences, 97% (72/74) contained the defining amino acid substitutions that characterize subclade K, including HA1:T135K, K189R that define parent subclade J.2.4, as well as the K2N, S144N, N158D, I160K, Q173R, and T328A markers unique to subclade K (1, 2).

**TABLE 1:** Number and percent of northeast Ohio influenza A virus (IAV) specimens sequenced and categorized by subclade and month of collection

			Month of year, 2024–2025, *N* (%)											
			Dec	Jan	Feb	Mar	Apr	May	Jun	Jul	Aug	Sep	Oct	Nov
Total IAV-positive samples			3,166	8,387	13,247	3,913	394	43	57	31	46	42	48	477
IAV samples sequenced^*a*^			7 (0.2%)	19 (0.2%)	7 (0.1%)	5 (0.1%)	13 (3.3%)	12 (27.9%)	16 (28.1%)	4 (12.9%)	10 (21.7%)	12 (28.6%)	7 (14.6%)	90 (18.9%)
A(H1N1)pdm09 or A(H1)	5a.2a	C.1.9	0	1 (5.3%)	0	0	0	0	0	0	0	0	0	0
		C.1.9.1	0	1 (5.3%)	0	0	0	0	0	0	0	0	0	0
		C.1.9.3	0	3 (15.8%)	1 (14.3%)	0	0	0	0	0	0	0	0	0
	5a.2a.1	D.3.1	0	3 (15.8%)	4 (57.1%)	5 (100%)	10 (76.9%)	11 (91.7%)	16 (100%)	3 (75.0%)	8 (80.0%)	8 (66.7%)	3 (42.9%)	22 (24.4%)
		D.5	0	2 (10.5%)	0	0	1 (7.7%)	0	0	0	0	0	0	0
A(H3N2) or A(H3)	2a.3a.1	J.2	7 (100%)	8 (42.1%)	2 (28.6%)	0	1 (7.7%)	0	0	0	0	1 (8.3%)	0	0
		J.2.2	0	0	0	0	1 (7.7%)	1 (8.3%)	0	1 (25.0%)	0	0	0	0
		J.2.4	0	0	0	0	0	0	0	0	2 (20.0%)^*b*^	0	0	1 (0.2%)^*c*^
		J.2.5	0	1 (5.3%)	0	0	0	0	0	0	0	0	0	0
		K	0	0	0	0	0	0	0	0	0	3 (25.0%)	4 (57.1%)	67 (74.4%)

^*a*^ All sequences have been submitted to GISAID (EPI_SET_251229gy).

^*b*^ Of the two A(H3N2) subclade J.2.4 sequences detected in August, one carried the HA1 mutations S144N, N158D, I160K, and T328A associated with subclade K but lacked K2N and Q173R, while the other had no HA1 mutations associated with subclade K.

^*c*^ This A(H3N2) subclade J.2.4 sequence detected in November had HA1 mutations S144N, N158D, I160K, and T328A associated with subclade K but not mutations K2N and Q173R.

These findings indicate that influenza A(H3N2) subclade K emerged and rapidly became predominant in northeast Ohio in the autumn of 2025. However, the study is limited by selection bias and the relatively few sequenced IAV-positive specimens collected prior to November 2025. Subclade proportions presented may not be reflective of the general population, and additional types and subclades may have been missed. Additionally, the possibility of unidentified reassorted viruses cannot be excluded due to incomplete segment coverage. The clinical significance of subclade K, including potential impact on disease severity and vaccine effectiveness, remains under investigation (3).

Our experience shows that IAV WGS implemented in a clinical laboratory can provide high-resolution genomic data that enhance public health surveillance. Near real-time sequencing was developed to enable IAV subtyping (8), but it has also enabled prompt identification of an emerging influenza subclade before traditional surveillance data for our region were available. This assay is now a clinically orderable test and currently being used to determine the IAV subtype for inpatients with influenza at our institution per Centers for Disease Control and Prevention (CDC) recommendations (8). As WGS becomes more common in clinical workflows, diagnostic laboratories are positioned to contribute timely information during periods of rapid viral evolution. Continued integration of genomic data with epidemiologic trends and vaccine effectiveness studies will be essential to understanding the impact of A(H3N2) subclade K as this influenza season progresses.
